# Multimodal Management of a Patient with Severe Mental Disorder: A Case of Escape and Re-admission to a Long-Term Psychiatric Care Unit

**DOI:** 10.1192/j.eurpsy.2025.1556

**Published:** 2025-08-26

**Authors:** R. Albillos Pérez, C. Peláez Fernández, S. Castelao Almodóvar, A. Castelao Escobar, P. Sanz Sánchez, C. Alguacil Núñez, R. González Núñez

**Affiliations:** 1 Psiquiatría, Hospital Universitario Puerta de Hierro Majadahonda; 2 Psiquiatría, Hospital Universitario El Escorial, Madrid, Spain

## Abstract

**Introduction:**

The follow-up of patients with severe mental disorders and disruptive behaviors poses a significant challenge, particularly when there is a history of high impulsivity and risk of escape. Effective coordination among mental health teams, support institutions, and families is essential to provide a safe therapeutic environment and facilitate rehabilitation. This case illustrates the complexity of managing a patient with conduct disorder and borderline intellectual functioning following a prolonged escape from a long-term psychiatric care center.

**Objectives:**

To describe the clinical and outpatient management of a patient who escaped from a long-term psychiatric care facility. To evaluate the importance of multidisciplinary and inter-institutional coordination in the planning and follow-up of treatment to optimize the clinical and functional stability of the patient.

**Methods:**

We present the case of a 26-year-old male with a history of conduct disorder and borderline intellectual functioning (IQ=60), problematic cannabis use, and gambling addiction. The patient was admitted to a Long-term Psychiatric Care Unit (UCPP) due to impulsive behaviors, aggression, and threats towards family members. The patient escaped and remained missing for 8 months. After being located and returning to the clinic, an outpatient intervention was initiated in coordination with the Madrid Agency for Support to People with Disabilities (AMAPAD) and the Regional Office of Mental Health and Addictions (ORSMA), assessing his readiness for re-admission and the adequacy of the treatment plan.

**Results:**

Upon re-admission, the patient initially appeared cooperative, minimizing the behaviors that led to his previous admission. As the intervention progressed, signs of irritability and hostility emerged, with overvalued/delusional ideas of persecution from family and UCPP staff, and limited introspective capacity. Intensive treatment at the UCPP led to better acceptance of limits and reduced impulsivity, although deficiencies in awareness of his limitations persisted. A transfer to another long-term care unit, the recently inaugurated Recovery and Community Reintegration Unit (URCC), was planned, with intensive rehabilitation goals tailored to the patient’s needs.

**Conclusions:**

Proper coordination among institutions and flexibility in the therapeutic approach are essential for managing patients with severe mental disorders and high-risk behaviors. This case underscores the importance of a multimodal approach and adapting therapeutic strategies to the patient’s clinical evolution, always prioritizing the safety and well-being of both the individual and their family environment.

**Disclosure of Interest:**

None Declared

